# Macrophages and their relevance in Human Immunodeficiency Virus Type I infection

**DOI:** 10.1186/1742-4690-9-82

**Published:** 2012-10-04

**Authors:** Herwig Koppensteiner, Ruth Brack-Werner, Michael Schindler

**Affiliations:** 1 Institute of Virology, Helmholtz Zentrum Munich, German Research Center for Environmental Health, Munich, Germany

## Abstract

Macrophages are important target cells for the Human Immunodeficiency Virus Type I (HIV-1) *in vivo*. Several studies have assessed the molecular biology of the virus in this cell type, and a number of differences towards HIV-1 infection of CD4+ T cells have been described. There is a broad consensus that macrophages resist HIV-1 infection much better than CD4+ T cells. Among other reasons, this is due to the presence of the recently identified host cell restriction factor SamHD1, which is strongly expressed in cells of the myeloid lineage. Furthermore, macrophages produce and release relatively low amounts of infectious HIV-1 and are less sensitive to viral cytotoxicity in comparison to CD4+ T cells. Nevertheless, macrophages play a crucial role in the different phases of HIV-1 infection. In this review, we summarize and discuss the significance of macrophages for HIV-1 transmission, the acute and chronic phases of HIV-1 infection, the development of acquired immunodeficiency syndrome (AIDS) and HIV-associated diseases, including neurocognitive disorders. We propose that interaction of HIV-1 with macrophages is crucial during all stages of HIV-1 infection. Thus, long-term successful treatment of HIV-1 infected individuals requires potent strategies to prevent HIV-1 from entering and persisting in these cells.

## Review

### Introduction

HIV-1 infects various cell types of the immune system. CD4+ T helper cells are major target cells for HIV-1 in the blood, since they can express high levels of the HIV-1 receptor CD4 on their surface and are highly permissive for HIV-1 production [1,2]. However, other immune cells also express CD4 and HIV-1 co-receptors at the cell surface and thus also serve as viral targets. Among them macrophages were described, more than twenty five years ago, to carry markers of productive HIV-1 infection *in vivo*[3], although they express only low levels of CD4.

Macrophages are terminally differentiated, non-dividing cells, derived from circulating monocytes [4]. They represent a distinct population of phagocytes which are found under different names in various tissues (e.g. microglia in the brain, alveolar macrophages in the lung, or Kupffer cells in the liver) [4,5]. Macrophages play an important role in the innate and adaptive immune response. They phagocytose cellular debris and pathogens, but also act as professional antigen presenting cells (APC), triggering antibody responses by the presentation of pathogen derived peptides via the MHC-II pathway to CD4+ T cells [5] and activating CD8+ cytotoxic T-cells (CTL) by cross-presentation of HIV-1 antigens [6]. The life spans of macrophages can differ greatly, depending on their immunological roles and tissue localizations. Thus inflammatory macrophages derived from circulating monocytes die after a few days [7], whereas microglia or alveolar macrophages can live from several weeks up to years [8-10]. Due to their dissemination over different tissues and their capacity to infiltrate virtually all organs including the brain, macrophages might critically contribute to the spread of HIV-1 within a patient [11-13]. Furthermore, next to human mammary epithelial cells [14,15], macrophages have been implicated as key cells responsible for mother-to-child transmission due to breast feeding [16].

The progressive loss of CD4+ T cells and high-level virus production by these cells are the irrefutable cause of immune deficiency [17]. However, the relevance of macrophages for the transmission, spread and pathogenicity of HIV-1 is less clear. One reason for this is the large diversity of possible interactions of macrophages with HIV-1. For example macrophages can differ both in their capacity to permit HIV-1 entry as well as their capacity to support the HIV-1 replication cycle [18-20]. Infection frequently results in only limited virus production, and *in vivo* infection may be apparent in only a minor proportion of macrophages within certain macrophage subpopulations [19,21,22]. In addition, macrophages are much more resistant to cytopathic effects of lentiviral replication than for example activated CD4+ T cells [23-25], and HIV-1 has evolved sophisticated mechanisms to prolong the life span of infected macrophages [24,26]. Especially long-lived macrophages may therefore harbor the virus for long time periods, thus constituting HIV-1 reservoirs and posing a major obstacle to virus eradication from infected individuals. Here, we summarize and discuss the growing body of evidence suggesting an important role of macrophages throughout the different phases of HIV-1 infection.

### Transmission of HIV-1 to the host: Macrophages encounter HIV-1 at mucosal surfaces

Worldwide, the predominant mode of primary HIV-1 infection is through heterosexual intercourse [27]. HIV-1 and other sexually transmitted pathogens have to pass the genital mucosal barrier, which strongly hinders infection due to its low pH, the closed epithelium and antiviral factors present in vaginal secretions [28,29]. Nevertheless, pathogens are able to cross the mucosal barrier, especially through small mucosal lesions that occur during sexual intercourse and impair epithelial integrity. Macrophages, dendritic cells (DC) and CD4+/CCR5+ memory T cells patrolling the mucosal surface are the first immune cells facing the virus [21]. Most sexually transmitted HIV-1 isolates use the CCR5 coreceptor for infection [30]. Therefore, next to CD4+/CCR5+ memory T cells, both dendritic cells as well as macrophages may be infected. Since mature DCs potently resist HIV-1 infection by various mechanisms, including the high expression of the recently identified restriction factor SamHD1, only a small proportion of DCs is productively infected [31-33]. Instead, they capture the virus via cell surface lectins such as DC-SIGN and home into lymph nodes or other secondary lymphatic organs, where they transmit surface bound HIV-1 to CD4+ T cells [32,34,35].

In contrast, resident macrophages in the mucosa usually do not migrate to lymph nodes. Non-infected macrophages take up and process the virus and present HIV-1 derived peptides via MHC-II to CD4+ T cells. Additionally, they help to optimize the anti-HIV CTL response due to cross presentation of virus derived peptides via MHC-I [6]. We postulate that cross priming of CTLs by macrophages and DCs is crucial for HIV pathogenicity, since an effective CTL response can control HIV-1 *in vivo*[36]. In addition it was recently demonstrated that HIV-1 infected macrophages can be killed by CTLs [37], although HIV-1 has evolved mechanisms to down-modulate MHC-I from the surface of virus infected CD4+ T cells [38,39] and macrophages [40,41]. Thus, macrophages in the mucosa contribute to the humoral and cellular immune response during the acute phase of HIV-1 infection.

A significant proportion of macrophages at the mucosal surface is productively infected with HIV-1 [42]. Since macrophages secrete cytokines that attract/recruit T lymphocytes to sites of infection, they can “support” establishment of viral infection by enlarging the number of primary target cells [43-46]. A particularly malicious feature of HIV-1 infected macrophages is that they may transmit the virus to CD4+ T cells at the mucosal surface via cell to cell contact during HIV-antigen presentation [47,48]. Considering the latter, we could think of a scenario in which a productively infected macrophage interacts with CD4+ T cells as a consequence of MHC class II mediated presentation of HIV-1 antigens and simultaneously transmits the virus to the interacting CD4+ T cell, even though this has not been shown experimentally, yet. The so-called virological synapse, which is established during cell-to-cell transmission of the virus [48,49], resembles the immunological synapse formed between antigen presenting cells and CD4+ T cells [50]. Thus, macrophage associated HIV-1 might hijack parts of the antigen presentation machinery for efficient transmission to adjacent cells.

In sum, recent evidence clearly establishes that vaginal macrophages are productively infected during sexual transmission of HIV-1. However, these tissue-associated macrophages stay at the mucosal surface and therefore probably do not transport HIV-1 to secondary lymphoid organs. Instead they recruit CD4+ T cells and contribute to the establishment of infection at sites of viral entry, i.e. the mucosal barrier.

### Hiking with macrophages: HIV-1 spread during the acute infection

During acute infection, virus is disseminated to secondary lymphoid organs, in particular to the gut associated lymphoid tissue (GALT). There is strong evidence that most of the CD4+ T cells in the GALT, including CD4+ memory T cells are directly depleted by massive HIV-1 propagation, accompanied by the loss of integrity of the intestinal barrier [51,52]. This causes translocation of lipopolysaccharide (LPS) and other bacterial products into the blood stream, driving generalized immune activation associated with rapid AIDS progression [51,53]. When compared to other macrophages like those of the vaginal mucosal tissue, intestinal macrophages seem to be relatively resistant against HIV-1 infection [42]. However, in their function as antigen presenting cells, they might be involved in the orchestration of the primary antibody response which suppresses HIV-1 virus loads at the onset of the chronic phase. Circulating monocytes are also recruited to the intestinal sites of viral replication and inflammation and differentiate into inflammatory macrophages. While these are permissive for HIV-1 infection [54], their role in the establishment and spread of HIV-1 is uncertain, since their half-life is only around two to three days [7]. Simultaneous to the establishment of infection in the lymphoid tissue and the breakdown of the intestinal barrier, virus is shed into the blood, which is evidenced by a dramatic increase in plasma viral loads [17,52]. From there, HIV-1 might infect perivascular macrophages, which have been shown in the monkey model to produce virus in the brains of SIV infected macaques, already at 14 days post inoculation [55]. In contrast to other tissue residing macrophages, perivascular macrophages are highly migratory and infiltrate other organs e.g. the lung and the brain [56]. They have a life-span of up to three months and are resistant to HIV-1 induced cytotoxic effects [57]. Hence, perivascular macrophages, next to latently infected monocytes, appear to be important cells for dissemination of HIV-1 throughout the body, including the brain. This conclusion is supported by evidence from studies by Thompson and colleagues, who detected viral DNA in perivascular macrophages and astrocytes in the brains of SIV infected macaques as early as 10 days post infection of the animals [58,59]. Furthermore the same group also demonstrated the presence of HIV-1 DNA in perivascular macrophages and parenchymal microglial cells as well as in astrocytes in human brain tissue of pre-symptomatic HIV-1 infected individuals that died of non-HIV associated reasons [59]. This finding raises the possibility of viral persistence in brain macrophages and astrocytes, which are both extremely long-lived cell types that may exist for the life span of the host [8,60,61].

Overall, macrophages play a two-faced role in the acute phase of HIV-1 infection. On the one hand, they help to establish infection at sites of viral entry and perivascular macrophages disseminate the virus in various organs including the brain. Thus, an important fact is that HIV-1 infection of the brain - an immune sanctuary and reservoir organ for HIV-1 - might occur early after HIV-1 transmission, during acute infection. On the other hand, macrophages are critically involved in the initiation and the orchestration of the adaptive cellular and humoral immune response which finally helps to diminish viral burden, leading to the reduction of viraemia which is typical of the onset of chronic infection [62].

### Macrophages are viral hideouts during the chronic phase of infection

The chronic phase can be considered as a standoff between the immune system and HIV-1. CD4+ T cells not only die because of cytotoxic T lymphocyte (CTL) responses but also due to active viral replication, direct HIV-1 induced cytotoxic effects, and excessive immune activation. However, depleted CD4+ T cells are replenished because of the regenerative capacity of the immune system and are prone to HIV-1 infection due to the generalized state of immune activation [63]. Cell free virus is efficiently inactivated by neutralizing antibodies; whereas cell-to-cell transmission enables HIV-1 to partly evade this immune response [64,65]. Thus, cell-to-cell transfer is likely to be the predominant mode of infection and spread in the chronic phase.

**Figure 1:**
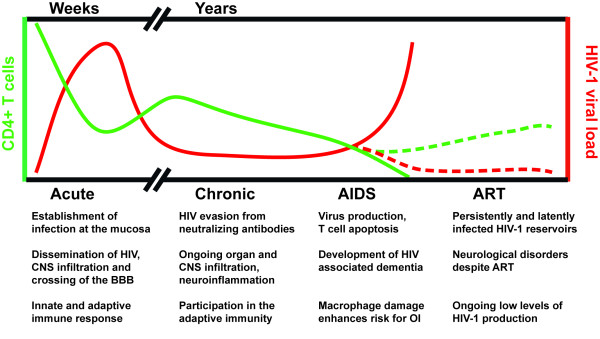
**Role of macrophages in HIV-1 infection and disease progression.** The number of CD4+ T cells and viral genome copies in plasma during the different phases of HIV-1 infection are presented in a schematic drawing. The dotted lines indicate the effects of antiretroviral therapy (ART). The contribution of macrophages to each phase of HIV-1 infection is indicated below the scheme. Abbreviations: CNS, central nervous system; BBB, blood brain barrier; OI, opportunistic infections; ART, antiretroviral therapy.

Accumulating data suggest that macrophages are important and specialized viral reservoirs, storing HIV-1 particles in internal compartments. The presence of mature HIV-1 in intracellular vesicles of macrophages was demonstrated long ago [66], and there is some controversy in the field regarding the origin of the HIV-1 accumulations in macrophages. This has been addressed in two other comprehensive reports [67,68] and is not the topic of the present review. Irrespective of the origin of intracellular virus containing compartments in macrophages, they seem to represent a hideout for HIV-1. The Stevenson lab demonstrated some years ago that macrophages can store infectious HIV-1 particles for many weeks [69]. Recently, we and others assessed the accessibility of the internal virus compartments from the exterior and found that high molecular weight substances, including broadly neutralizing antibodies are excluded [65,70]. Thus, infectious HIV-1 within macrophages generally seems to be protected from neutralizing antibodies. Of note, there are some hints that HIV-1 could rapidly be transferred from macrophage internal compartments to adjacent CD4+ T cells and uninfected MDM [48,49], and recently it was postulated that HIV-1 infected macrophages release virus containing exosomes and microvesicles to facilitate and enhance HIV-1 dissemination [71]. In sum, potent immune evasion mechanisms mediated by macrophages contribute to the inability of the immune system to achieve HIV-1 clearance within the acute and chronic phases of infection (see also Figure 1).

### The role of macrophages during AIDS progression

The continuous killing of CD4+ T cells in the course of HIV-1 infection inevitably leads to an impaired immune response, the acquired immune deficiency syndrome (AIDS). AIDS is characterized by a breakdown of the immune system and the loss of its capacity to control HIV-1 viraemia and to protect against opportunistic pathogens and tumors [17]. While mainly HIV-1 variants that use CCR5 as coreceptor (R5 viruses) are transmitted and prevalent during acute infection, a switch toward viruses that use the CXCR4 coreceptor (X4 viruses) occurs in about 50% of patients in the course of infection [72]. Since X4 viruses exert increased cytotoxicity, this coreceptor switch is associated with an accelerated progression of AIDS [73]. Macrophages are infected by CCR5 tropic HIV-1. This raises the question whether *de novo* infection of macrophages plays a subordinate role for AIDS pathogenesis. However, it has to be considered that the majority of CD4+ T cells are depleted in the AIDS stage; and a large proportion of patients progressing to AIDS still harbor viruses that use CCR5 for cell entry [72,74]. This indicates that macrophages indeed are involved in the late stages of HIV-1 infection.

Figure 1 depicts various reasonable scenarios for the potential relevance of macrophages for disease progression, some of which are supported by evidence from the SIV/monkey model. For one, HIV-1 infected macrophages might be responsible for a large proportion of the virus load in the face of declining CD4+ T cells [75]. In addition, since macrophages and monocytes are important cells for the orchestration of the innate immune response, macrophage-damage might impede the host defense against opportunistic infections [76-78]. In contrast, macrophages might also serve as targets for AIDS relevant pathogens, e.g. *Mycobacterium tuberculosis,* thereby fueling the establishment of opportunistic infections associated with the progression of AIDS.

Apart from the more obvious roles of macrophages in AIDS progression, there is a sophisticated regulation of macrophage activation and deactivation that could critically influence HIV-1 pathogenicity [46]. This concept of differential macrophage polarization in the course of AIDS progression was introduced by Guido Poli and can now be refined by recent progress in this area [79,80]. Blood circulating monocytes or monocyte-derived macrophages (M0) are either differentiated into proinflammatory M1 or anti-inflammatory M2 macrophages devoted to tissue repair. Macrophage polarization is influenced by a number of cytokines, however, mainly by GMCSF (M1) or MCSF (M2) [46,81]. Due to the high levels of MCSF circulating in the plasma, it is highly likely that the M2 phenotype generally prevails in the blood prior to acute HIV-1 infection. Interestingly, M2 macrophages restrict HIV-1 infection at a post-integration step without effects on protein expression levels or HIV-1 DNA [79]. In contrast, M1 macrophages restrict HIV-1 prior to integration, but do not exhibit a post-integration block [79]. Intriguingly, in the acute phase of infection, the Th1 response in conjunction with a number of proinflammatory cytokines favors the M1 phenotype of macrophages. Thus, previously infected macrophages with a M2 phenotype are now shifted toward a productively infected M1 profile [46,80]. The activation state of macrophages is highly flexible and might vary depending on tissue localization and specific macrophage functions [80,81]. This argues against postulating a general polarization state of macrophages in a certain stage of disease. Nevertheless, it has been proposed that the majority of macrophages in later HIV-1 infection stages represent IL-4/IL-13 activated M2 macrophages which would restrict HIV-1 production [46]. In this context it is noteworthy, that a recent report demonstrated a Nef-driven phenotypic shift of M2 to M1-like macrophages [82]. Thus, we could envision a scenario in HIV-1 infected patients, in which mainly M2-prevailing macrophages are infected. These M2-macrophages produce HIV-1 proteins, but no infectious virus particles, due to a terminal restriction that blocks completion of HIV-1 replication. However, this restriction is subverted by the Nef induced phenotypic shift to M1-like macrophages, resulting in completion of the viral cycle and a proinflammatory M1 phenotype.

Nef-activated and HIV-1 infected macrophages might be critically involved in the apoptosis of CD4+ and CD8+ T cells. While we could not confirm direct apoptotic effects of Nef in primary HIV-1 infected T cells [39], a more complex signaling network involving Nef in HIV-1 infected macrophages seems to be responsible for bystander T cell apoptosis. This whole concept is elaborated in a recent review by Herbein and colleagues and we refer the interested reader to this work for more details [45]. In sum, HIV-1 Nef, Tat and Vpr promote the survival and gene expression of HIV-1 infected macrophages. CD4+ T cells will be activated by factors secreted from infected macrophages and either killed directly, killed by infection or subverted into CD4+ T cell reservoirs. In contrast, CTLs are destroyed by a pathway dependent on the synergistic action of the TNF/TNFR and GP120. These complex mechanisms of CD4+ and CD8+ T cell death induced by HIV-1 infected macrophages will undoubtedly contribute to the total loss of T cells and finally AIDS development. With the appearance of opportunistic infections, macrophages will finally be deactivated due to increasing levels of IL-10 [46,81]. In the very late stages of disease, this will lead to a total breakdown of macrophage mediated adaptive immunity and immune deficiency.

### HIV-1 infected macrophages under antiretroviral therapy (ART)

Current ART involves treatment of infected individuals with several anti-HIV drugs that target different steps of viral replication. ART can permanently suppress viral loads in the plasma to levels beneath the detection limit of most assays approved for clinical use [83,84]. However, even long term ART cannot eliminate all infected cells from the patients, and virus levels rapidly rebound if antiviral treatment is stopped [83,85]. This is due to the persistence of HIV-1 in long-lived reservoirs. Cell types that contribute to HIV-1 persistence by formation of reservoirs include latently infected resting CD4+ T cells with integrated provirus, dendritic cells, macrophages, bone marrow haematopoietic stem cells and astrocytes [86-89]. Furthermore, persistence of HIV-1 is also promoted by the presence of the virus in so called viral sanctuaries – infection sites in the body which are difficult to reach by antiviral drugs and are additionally immune privileged niches [13,90]. Therefore, eradication of HIV-1 from these sites is hardly feasible [89]. One of the most important HIV-1 sanctuaries is the central nervous system (CNS), especially the brain. It contains HIV-1 reservoir cells, infected macrophages and astrocytes, for long-term virus persistence. This virus persistence is one of the major challenges for HIV-1 therapy and cure [61,91]. The presence of HIV-1 in reservoirs and sanctuaries leads to a dramatic increase of viral load if the therapy is stopped or interrupted, with the consequence that HIV-1 infected individuals require lifelong ART [92].

Macrophages are definitively involved in boosting HIV-1 rebound after stopping ART. Macrophages store high amounts of unintegrated viral DNA in circular form, and infected macrophages and monocytes were found in ART treated HIV-1 patients with viral loads under the detection limit [93-95] as well as in the brains of pre-symptomatic HIV-1 patients [59]. Furthermore, as already indicated, HIV-1 produced by tissue-associated macrophages might be targeted insufficiently by antiviral drugs due to the low bioavailability of the drugs in certain tissues [96]. Another remarkable feature rendering macrophage associated HIV-1 resistant toward HIV-1 protease inhibitors (PI) are multidrug pumps [97-99], although their involvement in PI resistance was recently questioned [100]. Their biological role is to allow macrophage resistance against toxins. However, these drug pumps also lower the concentration of inhibitors within the macrophage, decreasing concentrations of the anti-HIV drugs and possibly promoting the emergence of escape mutants [97,99]. Collectively, all these different lines of evidence establish that even under prolonged ART, HIV-1 persists in macrophages.

### Involvement of macrophages in HIV-1 associated neurological disorders

HIV-1 infection of the CNS/brain is associated with various nervous system dysfunctions. A severe form of neurocognitive impairment, called HIV-1 associated dementia (HAD), can occur in up to 10% of untreated individuals. While HAD has declined since the introduction of ART [101], a milder disease form, HIV-1 associated neurocognitive disorders (HAND), continues to prevail in up to 50% of HIV-1 infected individuals, even under optimal treatment conditions [102-104].

Monocytes and macrophages mediate HIV-1 neuroinvasion and contribute to HIV-1 in the brain and neuronal damage [12,13,105]. As already outlined, invasion of the brain begins very early in infection and can continue throughout the lifetime of the infected individual. Invading CD14+ CD16+ monocytes and perivascular macrophages can transmit the virus to microglial cells and astrocytes and establish infection and chronic inflammation of the CNS [106,107]. Neuroinvasion of HIV-1 may dysregulate the blood brain barrier and alter its permeability by various mechanisms including infection and loss of astrocytes [108].

Neurons very rarely show signs of HIV-1 infection *in vivo*, and entry and replication of HIV-1 in human neuronal cultures is very inefficient [109]. Thus glial cells, both microglia (brain macrophages) and astrocytes, are believed to be the effector cells of neuronal damage. Microglia and perivascular macrophages are the principal innate immune cells of the brain and therefore believed to play a central role in causing the neurological dysfunctions associated with infection [106]. Markers for productive HIV-1 infection (e.g. Gag proteins) have been identified mainly in macrophages in brain tissues from infected individuals, leading to the notion that macrophages are the predominant target cells for HIV-1 in the brain [110]. Furthermore, macrophage-tropic HIV-1 *env* genes and HIV-1 variants have been isolated from brain tissues of HIV-1 infected individuals, providing further support for HIV-1 infection of brain macrophages [74,111,112]. While the role of other brain cell types in HIV-1 infection and neuropathogenicity is beyond the scope of this review, it is important to point out that macroglia cells, particularly astrocytes, very likely also play important roles in HIV-1 entry, neuronal damage and virus persistence in the brain [109,113-115].

The histopathological hallmarks of HIV-1 infected brains include accumulation and activation of brain macrophages and occurrence of multinucleated giant cells, probably reflecting fusion of HIV-1 producing microglial cells [116]. The number and extent of activation of macrophages/microglial cells seem to be a better correlate for the severity of neurological disease cells than productive infection. Activation of monocytes/macrophages is mediated both directly by HIV-1 infection, and indirectly by factors secreted by activated cells in the brain. Activated macrophages/microglia can secrete a plethora of potentially neuromodulatory cellular factors, both harmful and protective [96,101,110]. Among them are TNF-α, IL-1β, IL-6 and macrophage colony stimulating factor (MCSF) as well as granulocyte monocyte colony stimulating factor (GMCSF). Once released, these cytokines can amplify the pool of activated cells and increase neuroinflammation of the CNS by paracrine and autocrine mechanisms [117,118]. Furthermore numerous studies have attributed neurotoxic activities to several HIV-1 proteins, including gp120, Nef, Tat and Vpr, which may occur both in cell-associated as well as soluble forms in the CNS (comprehensively reviewed in [46,101,109,119] for example). The aforementioned polarization of macrophages to an M1-like phenotype by the HIV-1 Nef protein might also contribute to neuropathogenesis [82]. M1 macrophages might be critically involved in the production and release of a variety of neurotoxic small molecules including quinolinate, platelet activating factor, nitric oxide and glutamate, all of which are involved in the development of neuronal injury, neuron and astrocyte death [101].

Clearly there is interplay between the different mediators of neuronal damage during HIV-1 infection of the brain, and this interplay makes it difficult to dissect the impact of an individual viral or cellular factor on HIV-1 induced neuronal damage. However, undoubtedly, macrophages play a central role in the development of HAND. Neurological disorders develop in all stages of HIV-1 infection, even under long term antiretroviral treatment [120]. Thus it will be of importance to further delineate the pathogenesis of HAND and develop antiretroviral and other drugs that are able to cross the blood brain barrier, target viral brain reservoirs and prevent HIV-1 production by microglia.

### HIV-1 restriction in macrophages

In recent years a variety of host cell factors suppressing HIV-1 at different steps in the viral replication cycle have been described and are now collectively called HIV-1 restriction factors (RF) [121]. Most but not all RFs are induced by interferon-α and exert potent antiviral activity in cell culture. However, despite the presence of host cell restrictions, HIV-1 efficiently replicates and causes AIDS in most untreated individuals. Coevolution of virus and host has resulted in the acquisition of potent viral antagonists that counteract viral restriction mechanisms of the host cell. For this purpose HIV-1 has a repertoire of versatile and rapidly evolving so-called accessory proteins, namely Nef, Vif, Vpr and Vpu. It is well established that Vif counteracts the cytidine deaminase APOBEC3G [122] and Vpu inactivates the antiviral factor Tetherin [123,124]. Furthermore, the Nef proteins of some simian immunodeficiency viruses (SIV) have evolved to block the action of Tetherin [125]. The SIV counterpart of Vpr, the Vpx protein, antagonizes the recently identified dideoxynucleotide hydrolase SamHD1 [33,126]. SamHD1 depletes the pool of deoxynucleoside triphosphates within the cell and thereby prevents reverse transcription of the HIV-1 RNA genome [127]. Of note, degradation of SamHD1 by Vpx is sufficiently rapid to allow reverse transcription post fusion of the virus with the host cell [128]. Since recent data suggest that counteraction of SamHD1 by Vpx is initiated in the nucleus [129], virion delivered Vpx has to be rapidly transferred into the nucleus before the initiation of reverse transcription.

Macrophages express high amounts of Tetherin and SamHD1, whereas CD4+ T cells express no or only low levels of these RFs [41,126,130]. As a consequence, in *ex vivo* experiments, viral production and release in macrophages is strongly impaired by tetherin and is only partly restored by Vpu nor does HIV-1 Vpr contain the ability to antagonize SamHD1 [41,126]. Other factors inhibiting HIV-1 replication in macrophages are for example Viperin [131] - although its general role in primate lentiviral restriction has recently been questioned [132] - and p21/cip/waf [133]. p21 might inactivate HIV-1 Integrase and therefore block efficient HIV-1 provirus formation [134]. However, p21 is broadly expressed and could also play an important role in non-myeloid cells [134,135]. Furthermore there is some controversy regarding p21 function since it may also be involved in post-integration regulation of viral transcription [87]. Other potent host cell restrictions in myeloid cells have been described [18], and recent exciting work identified novel restriction factors e.g. NMAPT/visfatin [136] and PAF1c [137] which might play previously unrecognized important roles in cells of the monocyte/macrophage lineage. Further experimentation investigating the role of the latter in the HIV-1 replication cycle is important and warranted.

What is the reason for efficient HIV-1 replication in macrophages *in vivo* despite the presence of RFs inhibiting replication *in vitro/ex vivo*? Not all RFs are counteracted by HIV-1 accessory proteins and for example Tetherin is expressed in very high amounts in macrophages [41,130]. As already outlined in this review, depending on their polarization and activation, macrophages are differentially permissive for HIV-1 infection and replication. Thus, macrophage polarization and activation could not only alter levels of HIV-1 restriction factors but also influence the expression of host genes positively regulating HIV-1 replication in macrophages, for example the recently identified proteins ADAM10 [138] and PKC-delta [139].

Macrophages can also restrict production of viral antigens from stably integrated replication-competent HIV-1 genomes, thus escaping detection by the immune system post infection. Various mechanisms may limit HIV-1 production in macrophages and other reservoir cells [87,91]. For example a mechanism recently discovered in astrocytes involves the selective restriction of production of viral structural proteins in cells with ongoing viral transcription by a family of host cell factors (Risp/Fam21) that interfere with the activity of the HIV-1 Rev protein [140]. Since viral structural proteins contain numerous antigenic epitopes [141], restriction of their production would facilitate the escape of the infected cell from the immune system.

Finally, there are large donor dependencies concerning the replicative capacity of HIV-1 in macrophages [142]. The constant coevolution of the virus and the host has not only shaped the functionality of viral accessory proteins but also of host cell factors, as was recently demonstrated for SIV Vpx and SamHD1 in a series of articles [143-145]. Considering this, host cell donor variations in macrophage RF expression levels, or polymorphisms in HIV-1 restriction factors affecting their functionality, might dictate the susceptibility towards HIV-1. In this context it is noteworthy that a recent report investigated the possible connection between SamHD1 polymorphisms in HIV-1 patients and infection and control of the virus. However, no association could be found [146]. Apart from this study, none of the hypotheses mentioned above, (i.e. correlation of host cell restriction factor expression in cellular subsets with virus loads and AIDS progression or potential correlation with viral RF countermeasures) has been experimentally challenged. Thus it will be of high relevance to answer them in future studies.

## Conclusions

In this review we have highlighted the tremendous importance of macrophages throughout all stages of HIV-1 infection (see Figure 1). Recent exciting developments include the discovery of novel restriction factors present in macrophages, the potential influence of macrophage polarization on the susceptibility towards HIV-1 and the cumulating experimental evidence establishing the role of macrophages in early HIV-1 transmission into the CNS, associated with neurological disorders. In consideration of the currently available potent therapy regimens that allow suppression of HIV-1 replication in HIV-1 infected individuals for decades, HIV-1 associated neurocognitive dysfunction will become an even more prominent problem in the upcoming years. Therefore, it is crucial to develop novel therapeutic options to target HIV-1 reservoirs in the brain. In addition, it might be indicated to treat HIV-1 already during acute infection in order to inhibit viral dissemination through infected monocytes and macrophages into the CNS and the formation of other long term reservoirs.

## Competing interests

The authors declare that they have no competing interests.

## Authors’ contributions

MS generated the initial manuscript draft and the figure; HK, RBW and MS contributed to writing and jointly developed the article to its final form. All authors read and approved the final manuscript.
